# Seroprevalence of Lawsonia intracellularis antibodies in intensive pig farms in China

**DOI:** 10.1186/1746-6148-10-100

**Published:** 2014-04-28

**Authors:** Zongxue Wu, Yong Ling, Deyu Tian, Qing Pan, Peter M H Heegaard, Cheng He

**Affiliations:** 1 Key Lab of Animal Epidemiology and Zoonosis of Ministry of Agriculture, College of Veterinary Medicine, China Agricultural University, Beijing 100193, China; 2 Innate Immunology Group, National Veterinary Institute, Technical University of Denmark, 1870 Frederiksberg, Denmark

**Keywords:** *Lawsonia intracellularis*, Prevalence, Antibody, Pigs, China

## Abstract

**Background:**

Porcine proliferative enteropathy caused by *Lawsonia intracellularis* (*L. intracellularis*) is a major concern to the pig industry worldwide. Although 8.3 billion pigs are produced each year in China, few reports on the prevalence of *L.intracellularis* infection are available. The aim of the current study was to estimate the seroprevalence of *L. intracellularis* antibodies in intensive pig farms in China.

**Results:**

A total of 1060 serum samples were collected from 14 commercial pig farms located throughout China. Animals from all age groups were sampled including pre-weaning piglets, weaners, fattening pigs, adult sows and boars. Antibodies against *L. intracellularis* were detected using a specific blocking ELISA. Of the 1060 serum samples, 602 were identified as positive using the ELISA test. The apparent seroprevalence of *L. intracellularis* seropositivity was 57% (95% CI 50 to 64%). The true prevalence (that is, prevalence corrected for the imperfect sensitivity and specificity of the testing method) was 77% (95% CI 70 to 83%).

**Conclusions:**

The highest true prevalence was observed in sows and boars, suggesting that within a herd these stock classes are a reservoir for infection. The prevalence of *L. intracellularis* seropositivity in local breed pigs was significantly less than that in imported breeds. A higher seroprevalence was found in pigs in herds in Central and Northern China, which may correspond to the greater use of the intensive production systems in these areas. We conclude that *L. intracellularis* is widely prevalent in commercial pigs in China.

## Background

*Lawsonia intracellularis* (*L. intracellularis*) is a gram-negative, fastidious obligate intracellular bacterium. The pathogen mostly occupies the small and large intestine in pigs and causes porcine proliferative enteropathy (PPE) post weaning. Infection leads to diarrhea, retarded growth and/or sudden death in fattening pigs. A high prevalence of *L. intracellularis* has been reported throughout the world, contributing to a substantial level of economic loss in the swine industry [1-3]. In some herds, the disease may manifest itself as severe hemorrhagic diarrhea with relative high mortality [4].

The first case of *L. intracellularis* infection in pigs was described in 1931 [5] and since that time, *L. intracellularis* has been reported in swine producing countries all over the world. In Denmark, 94% of tested animals were positive by PCR on feces [6], in Sweden 48% of herds were positive in fecal samples tested using nested PCR [7] and in Korea [8] 47% of herds were positive when fecal samples were tested using multiplex PCR. More recent reports indicated a 100% seropositivity in Korea [9], 91% in the USA [10] and 84% in Australia [11]. In Australia *L. intracellularis* has been estimated to cost the industry in the order of USD 25 per sow annually [12] and direct losses of USD 3 to 11 per affected animal [13].

Highly intensive management of domestic pigs is widely promoted in mainland China, where there is an estimated pig population of approximately 8.3 billion [14]. Severe diarrhea occurs frequently in fattening pigs and pregnant sows, having negative impacts on herd feed conversion rates and herd profitability. Importantly, *L. intracellularis* infection receives comparatively little attention from animal health authorities compared to highly pathogenic infections such as porcine reproductive and respiratory syndrome (PRRS), classical swine fever (CSF) and *Streptococcus suis* infection.

In 2008, the first isolate of *L. intracellularis* was identified from the intestinal mucosa of infected pigs in Southern China [15]. The apparent prevalence of *L. intracellularis* infected pigs identified by a PCR method was 14% and 16%, in weaners and finishers, respectively in Guangxi province, Southern China [15]. To the best of the authors’ knowledge the prevalence of *L. intracellularis* infection in pigs in other areas of China has not been reported. Moreover, the major transmission routes are unclear in different stock classes and production systems. In this study our aim was to determine the seroprevalence of *L. intracellularis* in pigs raised in the major pig-producing provinces in China. An additional aim of our study was to document the association between *L. intracellularis* positivity and the presence of diarrhea.

## Methods

A cross-sectional survey carried out between January and May 2011 to estimate the seroprevalence of *L. intracellularis* infection in Chinese pigs. Seven provinces (Beijing, Hebei, Tianjin, Henan, Hubei, Guangdong and Guanxi) took part in the study. Sampling was carried out using a two-stage cluster design. Two herds from each of the seven provinces were selected at random from a sampling frame of 55 intensive pig cooperatives listed by the Chinese Ministry of Agriculture. Sample size calculations were carried out to determine the appropriate number of individual pigs to be sampled from each of the selected herds. Previous reports have estimated the within-herd prevalence of *L. intracellulari* infection to be around 13% in weaning pigs and 16% in finishing pigs [15]. Based on a previous report [16], a credible estimate of the intra-cluster correlation coefficient for *L. intracellularis* infection was 0.06. We assumed an average cluster (i.e. herd) size of 80 pigs. Sample size calculations were carried out on the basis that we wanted to be 95% certain that our final estimate of *L. intracellularis* prevalence was within 5% of the true population value.

A total of 1060 serum samples were collected from pigs from the 14 herds that took part in the study. This included 147 sera from pre-weaning piglets (2 to 4 weeks of age), 221 sera from weaned piglets (5 to 7 weeks), 279 sera from fattening pigs (8 to 14 weeks), 255 sera from adult sows, and 158 sera from boars. Landrace and Large White (breeds exotic to China) and domestic breeds (Meishan and Jinhua) were represented in the sample. The pig numbers were 332, 287, 211 and 230 for Landrace, Large White, Meishan and Jinhua pigs, respectively. Serum samples were stored at −80°C until assayed.

To identify the association between *L. intracellularis* seropositivity and the presence of diarrhea, 659 sera were obtained from herds with animals having a recent history of clinical diarrhea while an additional 401 samples were collected from herds without a recent history of diarrhea.

All sera were tested for antibodies against *L. intracellularis* using a blocking ELISA purchased from Synbiotic Europe SAS (Lyon, France) according to the manufacturer’s instructions. The sensitivity and specificity of this test was 72% and 93%, respectively [17].

The apparent prevalence of *L. intracellularis* seropositivity was calculated using the number of pigs identified as seropositive divided by the total number of pigs tested. Apparent prevalence estimates were then converted to true prevalence estimates using the approach proposed by Rogan and Gladen [18]. The Rogan and Gladen approach was used in this situation (in preference to more complex Bayesian methods) because the apparent prevalence estimates were relatively high. Confidence limits for the apparent and true prevalence estimates were determined using the Wilson binomial approximation [19,20]. Confidence intervals for both the apparent and true prevalence estimates were adjusted using the design effect, a function of the intra-cluster correlation coefficient for *L. intracellularis* infection and the average cluster size. The significance of differences between the true prevalence *L. intracellularis* seropositivity for pigs in different groups (i.e. location, stock class and breed) was assessed by considering their confidence limits. If the confidence intervals for two groups did not overlap, the inference was that the two prevalence estimates were significantly different at the alpha level of 0.05 [21]. All the calculations were performed using the AusVet epiTools procedures [22].

This study was approved by the Ethical Committee for Animal Experiments at China Agricultural University, Beijing, China.

## Results

*L. intracellularis* antibodies were found in pigs of all ages. The true prevalence of *L. intracellularis* seropositivity was 77% (95% CI 70 to 83%). The true prevalence of *L. intracellularis* seropositivity ranged from 68-87% on stock class.

With respect to provinces, true prevalence of seropositivity varied, ranging from 43% (95% CI 22 to 64%) in Guanxi, Southern China to 100% (95% CI 100 to 100%) in Hubei, Central China. Overall, the true prevalence of seropositivity was significantly greater in Central China compared with Southern China and significantly greater in Central China compared with Northern China (Table 1).

**Table 1:** **Antibodies to *L. intracellularis *** **in commercial pigs by province and region**

**Province**	**Number of pigs**^**#**^		**Prevalence (95% CI)**^**†**^	
	**Positive**	**Tested**	**Apparent**	**True**
Northern China:				
Beijing	88	145	61 (41 to 80)	83 (68 to 98)
Hebei	87	146	60 (40 to 79)	81 (65 to 96)
Tianjin	75	146	51 (32 to 71)	68 (50 to 87)
Sub-total	250	437	57 (46 to 68)	77 (68 to 87)^a,b^
Central China:				
Henan	118	187	63 (46 to 80)	86 (74 to 98)
Hubei	137	188	73 (58 to 88)	100 (100 to 100)
Sub-total	255	375	68 (57 to 79)	94 (88 to 100)^a,c^
Southern China				
Guangdong	58	124	47 (25 to 68)	61 (40 to 82)
Guanxi	43	124	35 (14 to 55)	43 (22 to 64)
Sub-total	101	248	41 (26 to 55)	52 (37 to 67)^b,c^
Total	602	1060	57 (50 to 64)	77 (70 to 83)

^#^Number of pigs tested, number of pigs positive to the *L. intracellularis* ELISA test and apparent and true prevalence of *L. intracellularis* seropositivity, by province and region.

^†^Number of pigs seropositive to *L. intracellularis* per 100 pigs tested.

^a-a,b-b,c-c^True prevalence estimates with the same superscript significantly different at the alpha level of 0.05.

The prevalence of seropositivity varied by stock class, with higher prevalence estimates in older animals. The true prevalence of *L. intracellularis* seropositivity was significantly greater in boars, sows and fattening pigs compared with pre-weaning piglets and weaners (Table 2). The true prevalence in pre-weaning piglets did not differ significantly from that of weaned piglets.

**Table 2:** **Seropositivity to *L. intracellularis *** **in commercial pigs by stock class**

**Age class**	**Number of pigs**^**#**^		**Prevalence (95% CI)**^**†**^	
	**Positive**	**Tested**	**Apparent**	**True**
Pre-weaning	47	147	32 (14 to 50)	38 (19 to 57)^a^
Weaners	58	221	26 (12 to 40)	29 (15 to 44)^b,c,d^
Fattening	166	279	60 (46 to 73)	81 (69 to 92)^a,b,e,f^
Sows	186	255	73 (60 to 86)	100 (100 to 100)^a,c,e^
Boars	145	158	92 (81 to 100)	100 (100 to 100)^a,d,f^
Total	602	1060	57 (50 to 64)	77 (66 to 87)

^#^Number of pigs tested, number of pigs positive to the *L. intracellularis* ELISA test and apparent and true prevalence of *L. intracellularis* seropositivity, by stock class.

^†^Number of pigs seropositive to *L. intracellularis* per 100 pigs tested.

^a-a,b-b,c-c,d-d,e-e,f-f^True prevalence estimates with the same superscript significantly different at the alpha level of 0.05.

With respect to breed, the true prevalence of seropositivity was higher in Landrace (98%, 95% CI 94 to 100%) and Large White (96%, 95% CI 90 to 100%) pigs compared with the local Meishan (60%, 95% CI 44 to 76%) and Jinhua (37%, 95% CI 22 to 52%) breeds (Table 3). There were no significant differences in the true prevalence of seropositivity for pigs of Landrace and Large White breeds.

**Table 3:** **Seropositivity to *L. intracellularis *** **in commercial pigs by breed**

**Breed**^**#**^	**Number of pigs**		**Prevalence (95% CI)**^**†**^	
	**Positive**	**Tested**	**Apparent**	**True**
Landrace	234	332	70 (59 to 82)	98 (94 to 100)^a^
Large White	199	287	69 (56 to 82)	96 (90 to 100)^b,c^
Meishan	97	211	46 (30 to 62)	60 (44 to 76)^a,b^
Jinhua	72	230	31 (17 to 46)	37 (22 to 52)^c^
Total	602	1060	57 (50 to 64)	77 (66 to 87)

^#^Number of pigs tested, number of pigs positive to the *L. intracellularis* ELISA test and apparent and true prevalence of *L. intracellularis* seropositivity, by breed.

^†^Number of pigs seropositive to *L. intracellularis* per 100 pigs tested.

^a-a,b-b,c-c^True prevalence estimates with the same superscript significantly different at the alpha level of 0.05.

The true prevalence of *L. intracellularis* seropositivity in herds with a recent history of diarrhea was 0% in pre-weaning piglets and weaners compared to 100% in fattening pigs, sows and boars (Table 4). For the fattening pig, sow and boar stock classes the true prevalence of *L. intracellularis* seropositivity was greater in herds that reported a recent history of diarrhea compared with those that did not report diarrhea (Table 4). The confidence limits for the true prevalence of seropositivity in herds with and without a recent history of diarrhea did not overlap for all stock classes except for boars, indicating an association between *L. intracellularis* seropositivity and the presence of clinical diarrhea.

**Table 4:** **Apparent and true *L. intracellularis *** **seropositivity in pigs from commercial herds by stock class and diarrhea status of the herd**

**Breed**	**Number of pigs**		**Prevalence (95% CI)**^**†**^	
	**Positive**	**Tested**	**Apparent**	**True**
Diarrhea present:				
Pre-weaning	2	94	2 (0 to 9)	0 (0 to 0)^a^
Weaners	9	129	7 (0 to 18)	0 (0 to 21)^b^
Fattening	124	159	78 (62 to 94)	100 (100 to 100)^c^
Sows	130	157	83 (69 to 97)	100 (100 to 100)^d^
Boars	116	120	97 (89 to 100)	100 (100 to 100)
Sub-total	381	659	58 (49 to 67)	78 (71 to 86)
Diarrhea absent:				
Pre-weaning	45	53	85 (61 to 100)	100 (100 to 100)^a^
Weaners	49	92	53 (28 to 78)	71 (48 to 93)^b^
Fattening	42	120	35 (14 to 56)	43 (22 to 65)^c^
Sows	56	98	57 (33 to 81)	78 (57 to 98)^d^
Boars	29	38	76 (42 to 100)	100 (100 to 100)
Sub-total	221	401	55 (43 to 67)	74 (64 to 84)
Total	602	1060	57 (50 to 64)	77 (66 to 87)

^†^Number of pigs seropositive to *L. intracellularis* per 100 pigs tested.

^a-b,b-b,c-c,d-d^True prevalence estimates with the same superscript significantly different at the alpha level of 0.05.

## Discussion

In this study, using a blocking ELISA, the overall true prevalence of *L. intracellularis* seropositivity was 77% (95% CI 70 to 83%). A higher rate of seroprevalence was found in fattening pigs, sows and boars compared with pre-weaning piglets and weaners. Breeds exotic to China had a higher seroprevalence compared with domestic pig breeds. This is the first report on the infection of *L. intracellularis* across China. The above evidence indicates that *L. intracellularis* infection is widespread among intensive pig farms in China.

In this study, none of the pigs had been immunized with *L. intracellularis* attenuated vaccine, but a high seroprevalence was found in the collected serum samples indicating exposure to the bacterium. The apparent seroprevalence estimates in this study are comparable with those reported in other studies. Apparent seroprevalence was 90% for sows and 56% for fattening pigs in a study conducted in the USA [23], while growing pigs and fattening pigs were found to be 45% and 59% seropositive, respectively, in a Korean study using an immunofluorescent assay as the detection method [9]. In Australia all herds tested positive for *L.intracellularis*-specific antibodies and the mean within-herd prevalence of positive samples was 84% [11]. Variation in the sensitivity and specificity of the diagnostic methods used to detect the presence of *L.intracellularis* contributes to the different serological prevalence estimates reported in Europe and Asia [17,21].

Pigs reared in herds in Central China had true prevalence estimates that were higher compared with pigs reared in herds in the North and South of the country. We speculate that the reason for this finding is that higher seroprevalence rates are associated with intensive pig production units which are common in Central China. The apparent prevalence of *L. intracellularis* seropositivity was similar for sows and boars and markedly higher than that reported for similar stock classes in other countries. Adult sows with active infection are likely to transmit infection to suckling piglets and infected boars transmit pathogens to sows by artificial insemination. Based on a single assessment of a herd’s diarrhea status we note that the prevalence of seropositivity in herds with a recent history of clinical diarrhea was similar to that of herds without a recent history of diarrhea which would imply some latency of *L. intracellularis* in sows and boars. The high true prevalence of seropositivity in pre-weaning piglets and boars free of diarrhea does not exclude the possibility that diarrhea was caused by other pathogens. Pig diarrhea is caused by a number of pathogens and mixed infections, including *E. coli*, *Brachyspira hyodysenteriae*, *Lawsonia intracellularis*, and *Salmonella spp*. [24].

The high true prevalence of *L. intracellularis* seropositivity identified in this study can be explained by a number of factors. First, inappropriate use of in-feed antibiotics contributes to *L. intracellularis* infection. In some situations there is a risk of animals developing a resistance to antibiotics such as tetracyclines, sulfonamides, fluroquinalones and zinc bacitracin that are routinely used in feed formulations [25]. A recent study based on data collected from pig herds in Southern China reported that antibiotic excretion rates for sows was 48 mg/day, weaning piglets 19 mg/day, growing pigs 7 mg/day and finishing pigs 1.5 mg/day [26] indicating that the usage of antibiotics and therefore excretion masses are correlated with stock class. High *L. intracellularis* seropositivity is known to be associated with withdrawal of in-feed medication in fattening pig diets. In this study, the true prevalence of *L. intracellularis* seropositivity for fattening pigs (81%, 95% CI 69 to 92%) was significantly greater compared with pre-weaning piglets (38%, 95% CI 19 to 57%) and weaners (29%, 95% CI 15 to 44%). To some extent, a low true prevalence of *L. intracellularis* seropositivity reflects the presence of long-term treatment with antibiotics [9].

A second explanation for the high true prevalence of *L. intracellularis* seropositivity is that the presence of infection in breeding herds may arise from the introduction of breeding pigs into a herd without application of appropriate quarantine measures. Every year, thousands of European and American breeding pigs are purchased and imported into China by commercial pig farmers. In most situations the risk awareness of the introducing *L. intracellularis* infection is underestimated and clinical cases of *L. intracellularis* infection are often misdiagnosed by local veterinary authorities as well as the pig purchaser. The apparent prevalence of *L. intracellularis* infection in exotic breeding pigs ranged from 26% to 74% in previous reports [6,27]. One survey indicated that 56% of seropositive pigs in Korea originated from pigs born outside of Korea [9]. Recent reports note that *L. intracellularis* is spread between herds mainly through the purchase of infected pigs and replacement stock [27,28]. The importance of transmission by non-porcine vectors is unknown. Following experimental inoculation, histological lesions develop in laboratory mice, rats and hamsters, but not in sparrows or chickens [29].

In addition to the above, transmission from boars is likely to play a role in the spread of *L. intracellularis* both within and between herds. In this study, the true prevalence in boars could have contributed to infection rates in sows bred by artificial insemination. In the herds that took part in this study, sows were mated using boar semen without antibiotic treatment. The impact of this practice on between-herd spread of infection needs to be clarified.

## Conclusions

We conclude that the prevalence of *L. intracellularis* seropositivity is relatively high in Chinese pigs. The highest true prevalence was observed in sows and boars, suggesting that within a herd these stock classes are a reservoir for infection. The prevalence of *L. intracellularis* seropositivity in domestic pigs was significantly less than that in imported breeds. A higher seroprevalence was found in pigs in herds in Central and Northern China, which may correspond to the greater use of the intensive production systems in these areas.

## Competing interests

This work was supported in part by NSFC grant 31172305 and Ministry of Science and Technology (MoST) grant 2012AA101302-4 (Cheng He). The authors declare that they have no competing interests.

## Authors’ contributions

ZW, YL and QP contributed to the study design, evaluated the data, and drafted the manuscript. DT was responsible for the coordination of the work and collected blood samples. PH contributed to the revision and CH contributed to the study design, obtained the funding, and corrected manuscript. All authors have read and approved the final manuscript.
